# Anetoderma: an alert for antiphospholipid antibody syndrome^☆^^☆☆^

**DOI:** 10.1016/j.abd.2019.04.010

**Published:** 2019-12-18

**Authors:** Mariana Piraja Genta, Marilda Aparecida Milanez Morgado de Abreu, Gisele Alborghetti Nai

**Affiliations:** a Department of Dermatology, Hospital Regional de Presidente Prudente, Presidente Prudente, SP, Brazil; b Department of Pathology, Universidade do Oeste Paulista, Presidente Prudente, SP, Brazil

Dear Editor,

A rare disorder, anetoderma describes a disorder of elastic fibers of the skin, characterized by circumscribed areas of atrophy and hypopigmentation. It still has an unknown etiology and may be associated with genetic causes, autoimmune mechanisms, infectious diseases, or elastophagocytosis. In addition, prothrombotic phenomena and the presence of antiphospholipid antibodies have been observed in these patients.

This case report describes a patient with primary anetoderma and antiphospholipid syndrome (AS). In many cases of AS, anetoderma may be the first or the most evident manifestation of the disease. This association, however, is often overlooked, and the actions that could prevent an episode of thrombosis do not occur.

The case of a 43-year-old male patient from Taciba, São Paulo, Brazil, with skin lesions on the arms and trunk for three years, is reported. He was previously epileptic and had history of deep venous thrombosis one year previously. He made continuous use of phenobarbital and carbamazepine. The dermatological examination showed rounded, hypochromic plaques with atrophic appearance and central flaccidity, distributed throughout the dorsal region and arms bilaterally (Figure 1, Figure 2). The hypothesis of anetoderma was made and the biopsy showed disorganized collagen fibers and ruptured elastic fibers in the superficial and middle dermis, as well as a mild superficial perivascular lymphomononuclear infiltrate in the dermis (Fig. 3). Hemogram, electrolytes, and hepatic and renal function were normal. A laboratory investigation was performed for thrombotic disorders, with three antiphospholipid antibody reagents (anti-cardiolipin, lupus anticoagulant, and anti-β2-glycoprotein-I) and thus the diagnostic criteria for AS were met. Finally, erythrocyte sedimentation rate, C-reactive protein, and rheumatoid factor were normal; serologies for HIV, hepatitis, and VDRL were negative. The patient was referred to the rheumatology and hematology service where he remained under medical monitoring.

**Figure 1:**
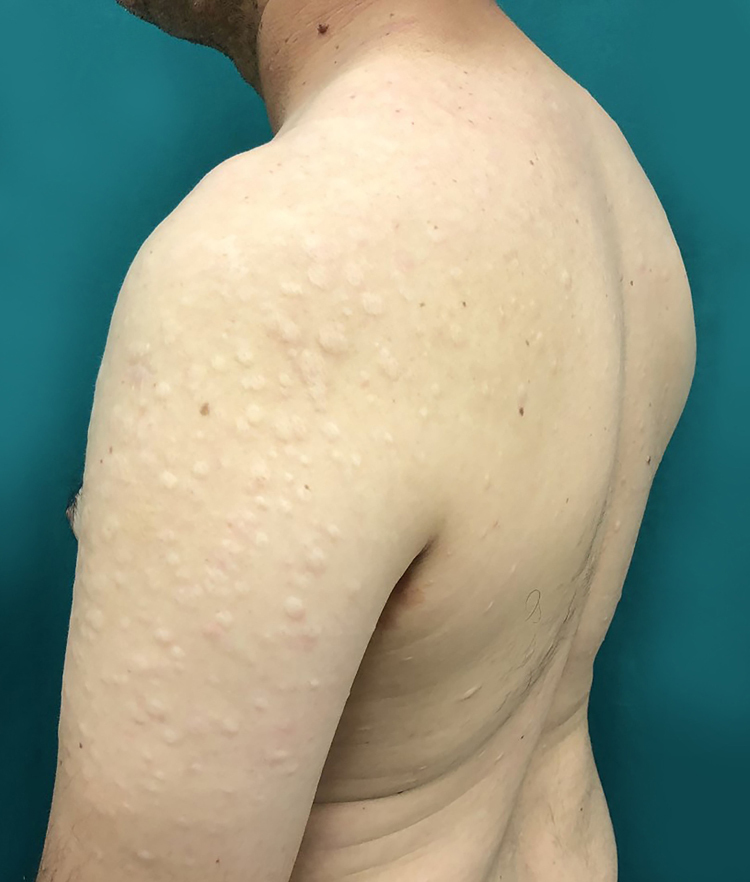
Multiple hypopigmented papules with central atrophy in the left arm.

**Figure 2:**
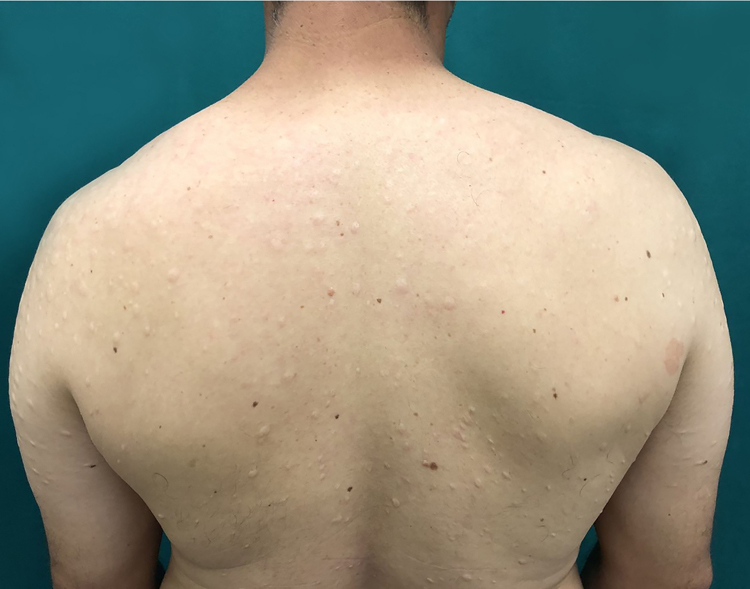
Multiple hypopigmented papules with central atrophy in the dorsum and upper limbs.

**Figure 3:**
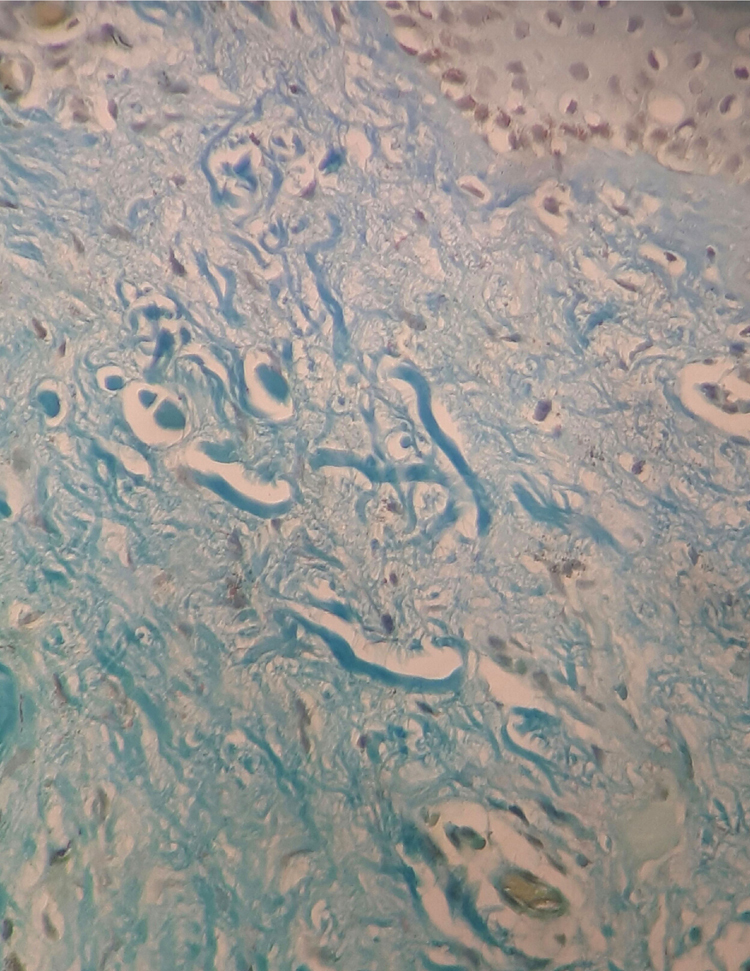
Shortened and fragmented elastic fibers in the papillary dermis (Verhoeff-Van Gieson, ×400).

Anetoderma is a disorder in which loss of elastic fibers occurs in the papillary or reticular dermis. It presents as papules or rounded, hypopigmented plaques with a subcutaneous hernia sensation. Its etiopathogenesis remains uncertain, and it can be classified according to the triggering conditions as primary or secondary. Primary anetoderma affects previously healthy areas of skin, since secondary anetoderma occurs in regions affected by previous pathologies, such as acne or varicella. Association with infectious processes, drugs, and autoimmune disorders, among them AS, have been reported as causes of primary anetoderma.^1^

AS is an acquired thrombophilia characterized by the presence of antiphospholipid antibodies, thrombotic disorders, and/or recurrent fetal loss. Complications with strokes are common and cutaneous involvement is also common. The cutaneous manifestations include the following: anetoderma, necrotic ulcers type livedoid vasculopathy, extensive necrotic ulcers (pyoderma gangrenosum), periungual ulcerations, necrotizing purpura, digital gangrene, multiple linear subungual hemorrhages, disseminated superficial cutaneous necrosis, livedo reticularis, livedo racemosa, acrocyanosis, bluish finger syndrome, chronic pigmented purpura, and chronic urticaria. Thus, anetoderma can be considered a cutaneous manifestation of AS.^2^

In the case reported, the patient had skin lesions compatible with anetoderma, which was confirmed by biopsy. Histopathology showed disorganized collagen fibers and ruptured elastic fibers in the superficial and middle dermis, and mild superficial perivascular lymphomononuclear infiltrate in the dermis. With the previous history of deep venous thrombosis in the lower limb, the possibility of AS was investigated and laboratory investigation was performed, evidencing antiphospholipid antibodies present and confirming the proposed diagnosis.

Recently, studies with patients with anetoderma showed that of nine patients affected, all nine had antiphospholipid antibodies and four met the criteria for the syndrome. In addition, of the nine patients studied, all had prothrombotic abnormalities. Therefore, the presence of an ischemic process followed by degeneration of the elastic fibers was suggested as the etiopathogenesis of the anetoderma in these cases.^3^

Other possible explanations described include the increase of gelatinases in the skin of patients with anetoderma, or the presence of a common epitope between elastic fibers and phospholipids (β2-glycoprotein1).^4^

There is no effective treatment for anetoderma. Although many modalities have already been used – intralesional injection of corticoid, penicillin G, salicylates, phenytoin, dapsone, and vitamin E – none of them has been proven satisfactory. In the described patient, intralesional corticosteroid injection was attempted, but without therapeutic response.^5^

Finally, this case highlights the importance of clinical investigation of coagulation disorders in patients with anetoderma, so that thrombotic events can be predicted and poor outcomes can be avoided in these patients.

## Financial support

None declared.

## Authors’ contribution

Mariana Piraja Genta: Conception and planning of the study; composition of the manuscript; critical review of the literature.

Marilda Aparecida Milanez Morgado de Abreu: Approval of the final version of the manuscript; conception and planning of the study; participation in the design of the study; intellectual participation in the propaedeutic and/or therapeutic conduct of the studied cases; critical review of the manuscript.

Gisele Alborghetti Nai: Collection, analysis, and interpretation of data; participation in the orientation of the study.

## Conflicts of interest

None declared.
